# AI in oral medicine: is the future already here? A literature review

**DOI:** 10.1038/s41415-024-8029-9

**Published:** 2024-11-22

**Authors:** Sultan Alotaibi, Eleni Deligianni

**Affiliations:** 41415188079001 Year 5 BDS Student, Division of Dentistry, School of Medical Sciences, FBMH, University of Manchester, UK; 41415188079002 Clinical Lecturer in Oral Medicine, Division of Dentistry, School of Medical Sciences, FBMH, University of Manchester, UK

## Abstract

**Objective** Artificial intelligence (AI) is reshaping many healthcare disciplines, mainly with newly developed computer systems or machines that have the ability to mimic human intelligence. This paper aims to review the available evidence on the applications of AI in oral medicine. The review critically assesses current evidence, shedding light on AI's growing role in this field.

**Methods** Around 20 applicable studies were included in this review from different databases like PubMed and Google Scholar. Studies included involved original research articles, mini-reviews, systematic reviews and meta-analyses.

**Results** Existing papers on AI uses in oral medicine included fundamental areas such as oral cancer, lichen planus, bisphosphonate-related osteonecrosis of the jaw, odontogenic keratocysts and oral lesions classification. AI has proved remarkable potential in terms of accuracy, sensitivity and specificity.

**Conclusion** The outcomes of the papers suggest that AI holds major potential to help dental practitioners diagnose and manage oral diseases with superior precision. While acknowledging the encouraging results, this paper also underscores the necessity for further research and improvement to fully harness the abilities of AI in oral medicine. It calls notice to the fact that AI, although a valued tool, should supplement rather than replace healthcare professionals.

## Key points

- Provides a comprehensive review of AI applications in various aspects of oral medicine, including the diagnosis and management of oral cancer, lichen planus, bisphosphonate-related osteonecrosis of the jaw, odontogenic keratocysts and the classification of oral lesions.
- AI systems have demonstrated high accuracy, sensitivity and specificity levels in diagnosing oral conditions.
- Emphasises the importance of addressing ethical considerations related to AI in healthcare, such as patient autonomy, accountability and biases in AI algorithms.
- Identifies a gap in AI education for dental professionals and suggests incorporating AI training into dental curricula and continuing education programmes.

## Introduction

In an era defined by technological developments, artificial intelligence (AI) has emerged as a revolutionary force that is reshaping many industries affecting our day-to-day life activities. AI generally refers to developing computer systems or machines that mimic human intelligence in undertaking certain actions.^1^^,^^2^^,^^3^^,^^4^^,^^5^^,^^6^^,^^7^ Russel^1^ describes how AI includes creating intelligent agents that can observe their environment and reasoning and how, through learning from experience, they can make judgements to attain certain targets. There are various subfields of AI, including machine learning (ML), computer vision, natural language processing and expert systems, which together allow machines to simulate and reproduce the cognitive abilities of a human being.^1^ Furthermore, deep learning (DL) is a fundamental subsection of ML which is capable of affording decision-making capacity and processing considerable data sets,^8^ wherein algorithms are organised to build artificial neural networks (ANN) with several hidden layers.^9^

AI has developed as a transformative technology in various fields of healthcare, improving disease diagnosis, treatment planning and patient management. A decisive application of AI in healthcare includes using ML in collecting patient data to improve clinical decision-making and eventually patient outcomes.^10^ Other benefits of AI include allowing healthcare professionals to connect with their peers worldwide,^3^ reducing time-consuming routine tasks,^6^ implementing a proper personalised management system of patients^11^ and the potential of performing healthcare services at a remote distance.^12^

Despite the growing interest in the development of AI in healthcare in recent years, and the many papers exploring its role in oral cancer (OC), the role of AI in oral medicine especially is still not clear. Therefore, the authors considered a literature review to comprehensively examine the existing publications on the role of AI in oral medicine practice and to the best of their knowledge, this is the first study reviewing this topic solely.

Overall, this paper aims to synthesise, analyse and evaluate the existing state of evidence related to the applications, benefits, challenges and future expectations of AI in the context of oral medicine. By reviewing the available research, we aim to provide insights into the efficiency and potential of AI-driven methods for the diagnosis, treatment planning and prevention of oral diseases and conditions. Moreover, the understanding gained from this review can potentially guide healthcare professionals, researchers and government officials in harnessing the power of AI to improve patient care.

## Methods and results

The review involves a comprehensive and inclusive search and selection method, applying robust methodology and pre-defined inclusion and exclusion standards. A preliminary search on Google Scholar alone using the term ‘artificial intelligence' yielded around five million results and adding ‘medicine' to it narrowed it down to three million. Finally, using ‘AI in oral medicine' in the search engine returned 300,000 results. Databases used were PubMed and Google Scholar based on availability and comprehensive numbers of published papers. Both authors independently conducted the search for relevant papers, reviewed the findings and reached a consensus on the studies to be included. As a result, around 20 applicable studies, including original research articles, mini-reviews, systematic reviews and meta-analyses, were identified and included. Languages of the papers were limited to English, Arabic, Greek, German and French, with no time restriction. Both authors independently extracted data from the included studies, which were synthesised, highlighting the key findings of using AI in oral medicine. The detailed characteristics of the studies included are summarised in Table 1.

**Table 1:** Table showing the characteristics of studies included

**Study ID**	**Authors**	**Year**	**Study title**	**Study type**	**Studies included**	**Sample size**	**Application in oral medicine**
1	Mahmood	2021	Artificial intelligence-based methods in head and neck cancer diagnosis: an overview	Review article	16	Not reported	Oral cancer
2	Kim	2022	Efficacy of artificial intelligence-assisted discrimination of oral cancerous lesions from normal mucosa based on the oral mucosal image: a systematic review and meta-analysis	Systematic review	14	Not reported	Oral cancer
3	Elmakaty	2022	Accuracy of artificial intelligence-assisted detection of oral squamous cell carcinoma: a systematic review and meta-analysis	Systematic review	16	6,606	Oral cancer
4	Alabi	2021	Machine learning in oral squamous cell carcinoma: current status, clinical concerns and prospects for future - a systematic review	Systematic review	41	61,330	Oral cancer
5	Hegde	2022	Artificial intelligence in early diagnosis and prevention of oral cancer	Systematic review	12	4,386	Oral cancer
6	Al-Rawi	2022	The effectiveness of artificial intelligence in detection of oral cancer	Systematic review	17	7,245	Oral cancer
7	Gomes	2023	Use of deep neural networks in the detection and automated classification of lesions using clinical images in ophthalmology, dermatology, and oral medicine - a systematic review	Systematic review	11	Not reported	Oral cancer
8	Mahmood	2020	Use of artificial intelligence in diagnosis of head and neck precancerous and cancerous lesions: a systematic review	Systematic review	11	Not reported	Oral cancer
9	Rajendran	2023	Image collection and annotation platforms to establish a multi-source database of oral lesions	Original research	N/A	2,474	Oral cancer
10	Jubair	2021	A novel lightweight deep convolutional neural network for early detection of oral cancer	Original research	N/A	716	Oral cancer
11	Patil	2022	Artificial intelligence in the diagnosis of oral diseases: applications and pitfalls	Review article	4	8,750	Oral cancer
12	Kar	2020	Improvement of oral cancer screening quality and reach: the promise of artificial intelligence	Review article	Not reported	Not reported	Oral cancer
13	Ilhan	2021	The contribution of artificial intelligence to reducing the diagnostic delay in oral cancer	Review article	Not reported	Not reported	Oral cancer
14	Paniti	2023	Artificial intelligence-based diagnosis of oral lichen planus using deep convolutional neural networks	Original research	N/A	1,089	Lichen planus
15	Chegani	2023	Artificial intelligence for rapid clinical diagnosis in oral medicine	Original research	N/A	1,089	Lichen planus
16	Kim	2018	Machine learning to predict the occurrence of bisphosphonate-related osteonecrosis of the jaw associated with dental extraction: a preliminary report	Original research	N/A	125	BRONJ
17	Rao	2021	Deep learning-based microscopic diagnosis of odontogenic keratocysts and non-keratocysts in haematoxylin and eosin-stained incisional biopsies	Original research	N/A	97	Odontogenic keratocyst
18	Gomes	2023	Use of artificial intelligence in the classification of elementary oral lesions from clinical images	Original research	N/A	5,069	Oral lesion classification

After a critical examination of the current literature, this review will encompass the following topics in oral medicine, including OC, lichen planus (LP), bisphosphonate-related osteonecrosis of the jaw (BRONJ), odontogenic keratocysts (OKC) and oral lesions classification.

## Oral cancer

OC is defined as malignant lesions involving the lips, hard palate, upper and lower gingiva, buccal mucosa, floor of the mouth and the anterior two-thirds of the tongue.^13^ Worldwide, OC accounts for 4% of all malignancies^14^ and ranks as the sixth most prevalent cancer.^12^^,^^15^ Most OCs are diagnosed at an advanced stage where the prognosis is poor, despite the substantial progress in understanding its pathogenesis, including the malignant transformation of oral potentially malignant disorders (OPMDs).^16^ Its early detection is key in the reduction of mortality and morbidity rates,^8^^,^^12^^,^^13^^,^^14^^,^^15^^,^^16^^,^^17^^,^^18^ plus improving survival rates to 75-90%.^19^ However, delayed diagnosis after metastasis has occurred drops the survival rates to less than 30%.^14^

Generally, diagnosing OC is based on the histological examination of the tissue taken from the patients through various sampling methods, including excisional or incisional biopsy, cytologic smears and fine needle aspiration.^16^ Biopsies are accompanied by certain limitations, including sampling errors leading to misdiagnosis, difficulty in locating the area of interest due to its non-uniform characteristics, and the shortage of qualified pathologists to evaluate intra-tumour heterogeneity.^17^ Furthermore, while histological evaluation of biopsies by an oral pathologist is still the accepted benchmark for OC diagnosis, it is prone to subjective interpretation due to variations in judgement and outcome inconsistencies.^20^ Also, despite the quick identification of advanced OC lesions, general practitioners still struggle with correctly identifying early-stage OC or OPMDs.^13^ Eventually, these will lead to delayed diagnosis, resulting in poorer prognosis, increased cost of care, and higher rates of morbidity and mortality.^13^ Consequently, alternative methods that provide more accurate and faster diagnosis are needed to improve patients' outcomes. Thus, recent growing interest in the use of AI in oncology has proven AI to be a useful tool in early detection of OC.^8^^,^^15^

Multiple systematic reviews have proven AI as a diagnostic aid for OC diagnosis.^14^^,^^17^^,^^18^^,^^21^^,^^22^ Elmakaty^17^ conducted a systematic review and meta-analysis examining the accuracy of AI in oral squamous cell carcinoma (OSCC) detection. Their findings support the ability of AI-assisted systems to diagnose OSCC with 92% sensitivity, 91.9% specificity, 11.4 positive likelihood ratio, 0.087 negative likelihood ratio, and 132 pooled diagnostic odds ratios. Some studies involved in the review demonstrated the ability of AI to predict lymph node metastasis and the five-year survival rate.^17^ Another paper by Kim^14^ reviewed the evidence on the effectiveness of AI-assisted systems in discriminating between oral cancerous lesions and typical mucosa by oral mucosal images. Different image tools assisted by AI were examined in the paper, including optical coherence tomography (OCT), autofluorescence and photographic images. OCT images were concluded to be the most accurate of all three, with 94% sensitivity, and photographic images and autofluorescence came then with 91% and 89% sensitivity, respectively.^14^ Furthermore, Alabi's^18^ systematic review assessed the use of ML in OSCC. A total of 41 studies fit their criteria in their review and the majority used a support vector machine (SVM) and ANN algorithms as ML techniques. Results showed 57-100% specificity, 70-100% sensitivity and 63.4-100% accuracy, which confirms the usefulness of ML methodologies in providing clinicians with a highly accurate approach, helping them make well-informed decisions.^18^ Additionally, Gomes'^21^ paper examined the use of deep neural networks in diagnosing and automatically classifying lesions through clinical images in the fields of dermatology, ophthalmology, and oral medicine. The review demonstrated performance like that of human specialists with regard to the prediction of survival of OC patients, which assists in early diagnosis and provision of treatment plans. Resources used with AI included autofluorescence, intra-oral probes, hyperspectral images, photodynamic therapy and computed tomography.^21^ Lastly, according to Mahmood,^22^ where they reviewed the ability of AI in detecting head and neck pre-cancerous and cancerous lesions, there is early evidence supporting the ability of supervised ML techniques in diagnosing and grading some types of OPMD. Even though most studies showed high accuracy, the quality of evidence was considered suboptimal, with an increased risk of bias that might have resulted in the elevated accuracy rates.^22^

Rajendran^23^ described in their research the ability of developing a platform for collecting images and annotating them in a worldwide image data set of oral lesions to help in automating lesion classification algorithms. A web interface was developed, which was hosted on a web server that collected oral lesion images from global cohorts, in addition to a customised annotation tool.^23^ They collected 2,474 images consisting of OC, OPMDs and other lesions on MimoSA UPLOAD and 800 of them were annotated by seven different oral medicine experts through MimoSA ANNOTATE. The paper concluded a moderate to high sensitivity (64.3-100%) in referral decisions for lesions that needed a transfer for cancer monitor, varying due to the type of the lesion. Such databases can accelerate the development of AI algorithms that could detect high-risk oral lesions early.^23^

Additionally, Jubair^4^ explained in their study the potential of developing a lightweight deep convolutional neural network (CNN) for the purpose of binary categorisations of oral lesions using real clinical images into ‘benign', ‘malignant', or ‘potentially malignant'. They used an approach involving a small deep CNN, using a pretrained EfficientNet B0 as a lightweight transfer learning model. In total, 716 clinical images were employed for training and testing the proposed model. The model achieved 85% accuracy, 86.7% sensitivity and 0.928 area under curve.^4^ Jubair^4^ concluded that applying deep CNNs offers an effective approach for building cost-effective embedded vision devices tailored for OC diagnosis, especially fit for scenarios with inadequate computational capabilities and memory resources.

Moreover, according to Patil,^15^ many papers have shown success in developing efficient AI models capable of diagnosing and predicting the recurrence of OC. One of which used ANN to differentiate between normal and pre-malignant tissues through laser-induced autofluorescence spectra copies, which resulted in 98.3% accuracy, 100% specificity and 96.5% sensitivity.^15^ Patil^15^ mentioned another study that used a CNN model to differentiate between the lesions from white light and autofluorescence images and concluded good diagnostic performance that can be improved with larger data sets. Interestingly, one study used DL to detect OC through confocal laser endomicroscopy images, resulting in 88.3% accuracy and 90% specificity.^15^

In addition, Kar^12^ believed that the application of AI in disease screening could boost diagnosis due to its augmented workflow and superior sensitivity when compared to conventional human-based screening methods, plus magnifying the scope of screening and improving the availability of early OC diagnosis. For instance, unlike traditional screening techniques, there is a lack of observational fatigue, and even minimal alterations within a single pixel range can be identified at a superior rate compared to human experts' observation.^12^

As early as 1995, AI was used to detect individuals with high OC risk.^13^ According to Ilhan,^13^ an affordable DL-reinforced smartphone-based OC probe with autofluorescence and polarisation images, plus combining them with OSCC risk factors by appropriate algorithms, can give specificities, sensitivities and positive and negative values between 81% and 95%. Hence, this system model can function as an economical and efficient technique to reduce delays within the professional and healthcare system, allowing patients to be prioritised for timely and appropriate treatment.^13^

All the studies mentioned have clearly proven AI's great potential in expediting OC diagnosis. But, several studies have used multiple ML models in the same paper which we believe could cause confusion, complexity and difficulty in interpretating the results. Also, we can notice how the quality of imaging tools and techniques used to train AI models can affect the precision of detection. We also believe that there was a reasonable amount of bias towards superior diagnostic results in some smaller studies where data were limited. Lastly, most studies have not considered the concerns and challenges of AI deployment in our daily lives thoroughly.

## Lichen planus

Clinical diagnosis of LP and differentiating them from other white, red and white-red lesions could be difficult for a general practitioner.^24^ As LP is an OPMD with a malignant conversion rate between 0.5-2.28%, it is crucial to detect it early.^24^ Thus, AI could be used in providing accurate and efficient detection of LP that outweighs conventional human-based diagnosis.^24^^,^^25^

Paniti's^24^ research aimed to apply AI via CNN to help differentiate between oral LP and non-oral LP in biopsy-proven cases. Their data included clinical photographs of 609 oral LP and 480 non-oral LP. All of these diagnoses have been confirmed histopathologically. All non-oral LP cases are of lesions usually mentioned in the differential diagnosis of oral LP, including hyperkeratosis, recurrent aphthous stomatitis, traumatic ulcer, lupus erythematous, erythematous candidiasis, carcinoma *in situ,* oral epithelial dysplasia, pemphigus vulgaris and mucous membrane pemphigoid.^24^ They only used 55 photographs selected at random from both groups as the test data sets, whereas the rest were used as validation and training data sets. The training data set undertook data augmentation to improve the amount and variety of images. The assessment of the CNN model's efficiency embodied performance metrics, such as accuracy, sensitivity, specificity, positive and negative predictive value, and F1 score. They concluded that CNN models can reach an accuracy of 82-88%.^24^ One can note that this study has used limited data and the idea of augmenting the data set could improve the outcomes and the paper's overall robustness.

Additionally, Chegani^25^ applied deep CNN on clinical images of LP. Their data set included 531 and 558 high- and moderate-resolution images of LP and mucocele, respectively. Images were taken by experts with intra-oral photography training and any images with disagreement to the diagnosis were discarded. They resulted in 84.9% recognition for LP and 76.02% for mucocele, achieved by Oromed AI software, the testing model used. Therefore, AI can act as a clinical adjunct in providing initial differential diagnosis.^25^

We believe that the effective use of AI in LP detection could help to avoid any healthcare costs caused by misdiagnosis since the exact source of LP is not fully understood. We also believe it is useful for researchers to explore the topic further and include lichenoid reactions, due to their evident similarity with LP clinically and histopathologically, as well as their acquired status as potentially malignant disorders.

## Bisphosphonate-related osteonecrosis of the jaw

Kim's^26^ research examined the ability of ML in predicting the incidence of BRONJ after tooth extraction. Patients included were treated in the oral and maxillofacial surgery department at Yonsei University College. Only patients with current or past bisphosphonate treatment for managing osteoporosis, in addition to undergoing tooth extraction, were included in the study. Exclusion criteria were patients with incomplete medical records, patients with a history of radiation therapy to the head and neck region, patients on bisphosphonates as part of cancer treatment, and any BRONJ cases not related to tooth extraction. A total of 125 patients fitted the criteria, comprising 84 controls and 41 cases. The authors used five different ML prediction algorithms, involving ANN, SVM, multivariable logistic regression model, random forest and decision tree. They concluded that random forest had the best results with 100% sensitivity and 83.3% specificity. The remaining ML models also achieved performance superior to traditional methods, with 81.8-100% sensitivity and 70.0-86.7% specificity.^26^

Even though ML has provided promising results in BRONJ prediction, it did not provide material on which type of bisphosphonates carries the greatest risk, the duration of drug holiday required and what the serum carboxy-terminal collagen crosslinks critical amount is.^26^ According to the authors, further parameters, for example, cause of tooth extraction, oral health, or certain gene expression, could enhance the models more. Also, due to the inadequate total and characteristics of patients, the researchers of the study regard it as preliminary.^26^

We believe that further investigation is needed to consider additional variables, such as if there is a specific tooth that increases the risk of BRONJ further, if complications occurring during the procedure affect the incidence rates, and whether the prescription of pre-operative antibiotic prophylaxis or suturing the socket make any difference.

## Odontogenic keratocysts

OKCs are rare cysts affecting the jaw, which represent 3-11% of jaw cysts and are considered as the third most common in India.^27^ OKCs are locally destructive cystic lesions leading to jawbone damage and root resorption of the tooth involved.^27^

Rao's^27^ paper examined the ability of DL-based microscopic detection of OKC and non-OKC in haematoxylin and eosin-stained jawbone cyst sections. The authors collected a total of 2,657 microscopic images at 400x magnification, consisting of 54 OKCs, 23 dentigerous cysts and 20 radicular cysts. Then, the images were methodically annotated by a pathologist and sorted into categories, involving the epithelium, cystic lumen and stroma of both keratocysts and non-keratocysts. After that, pre-processing was done in a two-step approach: first, data were augmented to improve the functioning of DL techniques through added data volume; and then the epithelial area was selected as the designated region of interest. The experiment yielded a notable accuracy of 93% for differentiating between OKC and non-OKC images. Also, when CNN was instructed on little patches of epithelium that were hand-crafted and then on the full image, a greater accuracy of 98% was obtained due to the huge dataset of 1,704 OKC cases and 1,635 non-OKC cases. The authors advise that the projected algorithm holds prospective for integration into the automation system of OKC and non-OKC detection. Furthermore, the application of a whole slide imaging scanner for image possession from slides offers the ability to improve accuracy rates and cut human bias.^27^

Even though this paper has shown promising results, we think that the system used is expensive and not sustainable for application in various regions of the world, especially developing countries. Also, the authors could further investigate the ability of AI to predict the recurrence of OKCs and if it could detect cysts in different parts of the body.

## Oral lesions classification

Differentiating between the characteristics of elementary oral lesions quickly makes things easier for healthcare professionals. Gomes^28^ conducted a study applying CNN to automatically categorise oral lesion images into categories, including papule, macule, vesicle, erosion, ulcer and plaque. The authors used 5,069 clinical images of oral lesions. The summary of the sensitivity, specificity and accuracy concluded for each category is: papule - 79.79%, 94.58%, 92.14%; macule - 94%, 100%, 98.99%; vesicle - 100%, 99.59%, 99.66%; erosion - 79%, 99.79%, 96.32%; ulcer - 71.71%, 95.19%, 91.30%; and plaque - 87%, 93.17%, 92.14%, respectively.^28^ High sensitivity and specificity were noticed in the following categories: vesicle, erosion, plaque and macule, compared to high sensitivity and medium specificity for the remaining two classes.^28^

Though this seems like a promising research paper, it appears that the authors have not considered the ability of lesions to have more than one pattern, which might be the case in LP lesions for example. Also, it is important to note that some images used in the study had low resolution or focus problems that might have affected the results obtained.

## Prospects and challenges

The rapid advancements of AI have paved the way for transformative visions in dentistry, promising to redefine various fields of it. For example, AI models trained appropriately can act as virtual dental assistants, providing administrative services like arranging appointments, managing dental insurance and revising full records of patients.^3^ However, just like any evolving technology, AI arrives with its own set of limitations that demand further exploration and vigilant regulation for efficient management. These boundaries underscore the difficulty of integrating AI into diverse sectors of healthcare. Ethical issues that may arise due to AI use include patient-informed consent to use their data, security and transparency, fairness and avoiding bias in the algorithms, and data privacy.^5^^,^^18^^,^^20^ Since healthcare data are usually complicated, confidential and liable to rigorous privacy regulations, researchers and instructors must collaborate with healthcare professionals, data experts and official bodies to guarantee that the data used to train algorithms of an AI model are obtained ethically and precisely.^11^ Therefore, to prevent patients' data leakage or its inappropriate use, we must implement comprehensive federated guidelines and appropriate legal frameworks.^15^ Also, to ensure ethical AI development and deployment, strategies such as inclusive data collection, transparency, regulatory compliance, continuous monitoring, interdisciplinary collaboration, and patient engagement are essential. These measures will help ensure that AI systems in oral medicine are used responsibly and equitably, ultimately benefiting patient outcomes and maintaining trust in clinical practice. Moreover, for AI to be accurate, it requires a huge database of high-quality data to interpret information precisely.^3^^,^^11^^,^^15^ Furthermore, if an AI model was used on clinical images, the quality of the image may vary greatly depending on many factors, like camera type and lighting, which may affect the interpretation leading to misdiagnosis.^14^ Moreover, using AI without any human supervision might expose vulnerabilities in cyber security.^5^ Lastly, the development of AI should do no harm to patients,^7^ and if so, who will take responsibility if the system fails?^18^^,^^20^

Additionally, a crucial factor in ethical consideration regarding AI applications in various fields of healthcare is respecting patients' autonomy. Patients should have the right to make informed decisions about their healthcare, and the use of AI should not undermine this principle. Transparent communication about the role of AI in diagnosis and treatment is essential. Patients should be informed about how AI algorithms work, the data they use and the implications of AI-driven decisions. Ensuring that patients understand and consent to the use of AI in their care is crucial for maintaining trust and autonomy.

It appears that AI, in the not so far future, will help healthcare professionals by allowing them to make well-informed decisions; however, it should always be up to the professional to decide if the results AI is giving them are accurate or false. Also, AI is trained using data from the latest guidelines and guidelines do not fit all patients' profiles. Moreover, guidelines change overtime. Though the latter can easily be addressed as new guidelines can be ‘fed' into the system, there are still considerations, as AI so far is unable to overcome clinical experience. Furthermore, there is always the question of new technology like this not only being effective but also being accessible to all parts of the world, so its benefits are maximised.

## Conclusion

In the final analysis, even though current evidence has proven AI to be effective and efficient, it is still considered to be at its infancy stage, and we can expect to see its influence growing in the upcoming years in various fields of healthcare, especially dentistry, resulting in better care for patients. Hence, incorporating AI education into dental programmes and continuing education programmes is vital for preparing dental professionals to effectively use AI technologies in dentistry. By providing foundational knowledge, practical training and education on ethical and legal considerations, dental schools and professional organisations can equip future and current dentists with the skills needed to embrace AI. This educational approach will facilitate the adoption of AI in oral medicine, ultimately enhancing patient care and advancing the field. However, we still believe that AI cannot replace humans, especially doctors and healthcare professionals, where the application of clinical judgement and experience is mostly needed.

## Data Availability

All data in this literature review were accessed through publicly available databases, including Google Scholar and PubMed. These sources provided the necessary research articles and relevant information analysed in this review. The data supporting the findings of this study are derived from these publicly accessible databases, and any additional data or materials related to this review can be obtained from the corresponding author upon reasonable request.
